# Identification and antigenicity of the *Babesia caballi* spherical body protein 4 (SBP4)

**DOI:** 10.1186/s13071-020-04241-9

**Published:** 2020-07-22

**Authors:** Mona S. Mahmoud, Omnia M. Kandil, Nadia T. Abu El-Ezz, Seham H. M. Hendawy, Bassma S. M. Elsawy, Donald P. Knowles, Reginaldo G. Bastos, Lowell S. Kappmeyer, Jacob M. Laughery, Heba F. Alzan, Carlos E. Suarez

**Affiliations:** 1 Parasitology and Animal Diseases Department, National Research Center, Dokki, Giza, Egypt; 2 Department of Veterinary Microbiology and Pathology, College of Veterinary Medicine, Washington State University, Pullman, WA USA; 3 Animal Disease Research Unit, United States Department of Agricultural-Agricultural Research Service, Pullman, WA USA

**Keywords:** Equine piroplasmosis, *Babesia caballi*, iELISA, SBP4, Serodiagnosis

## Abstract

**Background:**

The tick-borne intra-erythrocytic apicomplexan *Babesia caballi* is one of the etiological agents of equine babesiosis, an economically important disease of equids in most tropical and subtropical areas of the world. Discovering candidate antigens for improved diagnostic tools and vaccines remains needed for controlling equine babesiosis. This study describes the *B. caballi sbp4* (*Bcsbp4*) gene and protein (*Bc*SBP4) and analyzes its antigenicity in infected equids.

**Methods:**

BLAST searches of an uncurated *B. caballi* assembly genome using the *B. bovis* SBP4 as a query were carried out, followed by PCR amplification and sequencing of a newly identified *Bc*SBP4. Characterization of this novel gene and protein was performed by bioinformatics analysis, western blots, immunofluorescence (IFA) and an *in vitro* neutralization test using anti SBP4 peptide antibodies. Antigenicity of recombinant *Bc*SBP4 (r*Bc*SBP4) was tested with sera from field animals (*n* = 18) using an indirect ELISA (iELISA).

**Results:**

*Babesia caballi* genome searches using *B. bovis* SBP4 as a query allowed identification of a novel gene termed *Bcsbp4.* The *Bcsbp4* gene encodes for a protein of 30.58 kDa, which is fully conserved among *B. caballi* isolates from USA and Egypt. Bioinformatics analysis indicates that *Bc*SBP4 contains a signal peptide and lacks additional transmembrane domains. Expression of *Bc*SBP4 in blood stages of *B. caballi* was confirmed by western blot and IFA using antibodies against synthetic peptides representing putative B-cell epitopes of *Bc*SBP4 predicted by *in silico* analysis. *In vitro* neutralization tests using anti-*Bc*SBP4 peptide antibodies showed a marginal, but statistically significant inhibitory effect on the infectivity of *B. caballi* merozoites in horse red blood cells. Sera from eight *B. caballi*-infected equids, but none out of ten negative equid control sera, gave a positive signal in an r*Bc*SBP4 based iELISA.

**Conclusions:**

The *Bcsbp4* gene is expressed in *B. caballi* blood stages. The *Bc*SBP4 protein is a potential candidate for developing a novel serological test that could detect *B. caballi* infection in equids in tropical and subtropical countries worldwide.
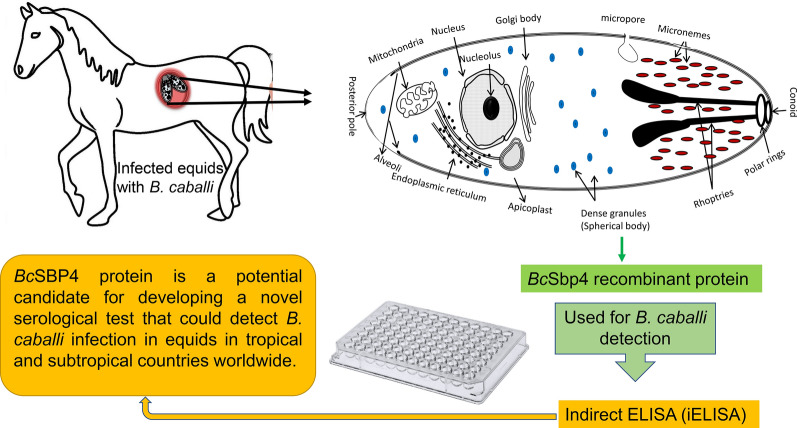

## Background

Domestic and wild equids, such as horses, donkeys, and zebras, remain important participants in numerous economic, cultural, and recreational activities in many countries worldwide. Furthermore, horses and donkeys are still heavily used and considered as valuable assets for transportation and other important agricultural activities in many developing countries, including rural areas in Egypt. Equine piroplasmosis (EP) is an economically important infectious tick-borne disease of Equidae currently listed by the “Office International des Epizooties” (OIE) that severely limits equid production and wellbeing. EP is endemic in most tropical and subtropical areas of the world and is caused mainly by two intra-erythrocytic haemoprotozoan parasites, *Theileria equi* and *Babesia caballi*. These parasites are transmitted by ixodid tick vectors [1, 2]. Both *T. equi* and *B. caballi* parasites are responsible for severe hemolytic disease that is characterized by fever, anemia, haemoglobinuria, jaundice, edema, and occasional death of the infected equids [3], which results in great economic losses in the horse industry in endemic areas. Clinical signs of EP caused by *B. caballi* are similar to *T. equi* and other hemoparasitic diseases of equidae, making it difficult to differentially diagnose solely on the basis of clinical presentation of the disease [4]. In addition, vaccines against *B. caballi* are currently unavailable, and control of the disease depends largely on accurate diagnostics and treatment with babesiacidal drugs, such as imidocarb. Although these treatments ameliorate the clinical signs and reduce fatalities [1], efficient control of *B. caballi* transmission would require sensitive and specific serological and molecular diagnostic methods.

The incidence of equine piroplasmosis in Egypt currently remains unknown. The lack of effective and practical diagnostic tools prevents large scale surveys, and the prevalence of *B. caballi* infection in Egypt remains mostly unknown, creating a critical knowledge gap on EP research and control. Furthermore, Egypt has many regions that provide a favourable environment for the development of competent tick vectors. Ticks remain uncontrolled, and it is possible that their habitats are expanding due to climatic change and human activities. Consistently, previous work based on small scale survey, using PCR, immunofluorescence (IFA) and ELISA techniques, suggested that infection of equids with *B. caballi* and *T. equi* is highly prevalent in Egypt [5, 6]. However, the small-scale serological survey performed by the National Research Center (NRC) [6] was in part based on a previously standardized and validated competitive ELISA (cELISA) test that had low sensitivity [7]. The cELISA used in these studies relies on the use of a monoclonal antibody (mAB) (Bc48 79/17.18.5) that recognizes an epitope expressed in a member of the *B. caballi* rhoptry-associated protein-1 (RAP-1) family [8]. Although the test demonstrated adequate sensitivity and specificity in previous studies [8], it was not able to detect the presence of specific antibodies against *B. caballi* Egyptian isolates in *B. caballi*-infected donkey and horse populations [6]. Consistently, the cELISA was also reported lacking in the ability to detect specific antibodies of *B. caballi* isolate in South Africa [9] and Israel [10]. Further analysis demonstrated that South African and Israelian *B. caballi* strains have significant polymorphisms in their RAP-1 sequences, including the B-cell epitope defined by the mAb 79/17.18.5 used in the RAP-1 based c-ELISA test [9, 10]. Taking this information together, it is clear that further investigations are urgently needed to identify alternative specific antigens that could be used for the development of novel methods for the serological detection of *B. caballi* infections in the Middle East and Africa which may also be applied worldwide.

The spherical bodies are organelles present in *Babesia* parasites and are believed to be the functional equivalent of the dense granules, a type of specialized secretory organelles that were described in other related members of the apicomplexan phylum [11–14]. Spherical body proteins (SBPs), which are secreted by the spherical bodies, are considered to have critical functions, including parasite-host cell interaction and immune evasion [11–13]. Hence, SBPs play a crucial role in the post-erythrocyte invasion steps, likely assisting in the growth and egression of infectious merozoites [13]. The SBP4, initially identified in *B. bovis*, is conserved among different geographical isolates and has shown an excellent performance when used as a serological antigen in several distinct ELISA formats for the detection of bovine babesiosis [14, 15]. Importantly, it appears that, at least in *B. bovis*, SBP4 is produced and secreted in a steady fashion throughout the parasite life-cycle in the vertebrate host, thus causing continuous and intense immunological stimulation during infection [14].

Hereby we identified a gene encoding for a SBP4 protein in the *B. caballi* genome termed *Bcsbp4*. We demonstrate that *Bc*SBP4 is expressed in blood stage parasites and immunogenic in *B. caballi*-infected equids. We also showed that *Bcsbp4* gene is highly conserved among the American and Egyptian *B. caballi* isolates. These features prompt us to propose the use of *Bc*SBP4 as an antigen to develop new diagnostic assays for EP caused by *B. caballi.*

## Methods

### *In silico* identification of the *Bcsbp4* gene and bioinformatics characterization

The *Bcsbp4* gene was identified in an uncurated genome assembly of *B. caballi* using the sequence of the *B. bovis sbp4* gene as a query in tBLAST using CLC Genomics Workbench software (Qiagen, Hilden, Germany) to create a database of genome assemblies and to BLAST against them. The predicted amino acid sequence of *Bc*SPB4 is shown in Additional file 1: Data set S1. Trans-membrane domains and signal peptides were predicted using the Transmembrane Hidden Markov Model package 2 (TMHMM2) (http://www.cbs.dtu.dk/services/TMHMM-2.0). For protein visualization MINOU (http://minnou.cchmc.org/) and PROTTER software were used (http://wlab.ethz.ch/protter). Sequence identities and similarities were calculated using online software in the website https://www.bioinformatics.org/sms2/ident_sim.html. The identity of the genes flanking the *Bcsbp4* gene was kindly provided by Lowell S. Kappmeyer and Dr Joana Silva, from the School of Medicine (University of Maryland, USA) and the Institute of Genome Sciences (Baltimore, USA).

### Collection of field equid blood samples

A total of 18 blood samples from apparently clinically healthy equines (8 horses and 10 donkeys) were collected from the Police Academy and the El-Giza zoological garden abattoir, both institutions are located in the city of Cairo, Egypt. Serum was collected as previously described [6]. Blood collected in EDTA-containing tubes was used for preparation of blood films for hematological evaluation and for DNA extractions using FTA^®^ Elute cards (Whatman, Buckinghamshire, UK). Blood films were stained with Giemsa stain according to manufacturer’s instructions. The stained blood films from the 18 donor equids used in this study were inspected under light microscopy for the presence of intraerythrocytic parasites.

### DNA isolation and PCR analysis

Total DNA was extracted from *B. caballi*-infected blood using FTA^®^ elute cards and purification reagent (Whatman) following the manufacturer’s instructions. DNA was eluted and PCR was performed for amplification of the *B. caballi sbp4* according to Mahmoud et al. [6] using primers Bcsbp4-For (5′-ATG GCT GCC TTC TCG ACC CGC TCC-′) and Bcsbp4-Rev (5′-CTC AGA CTT TTC GGC GGC TTC AGC-3′). The sequences of the primers were derived from the *sbp4* gene identified in the *B. caballi* genome. *Babesia caballi* DNA positive control for PCR was kindly donated from the OIE equine piroplasmosis reference laboratory located in Pullman, WA, USA. The PCR amplicons were electrophoresed on 1.5% agarose gel and stained with SYBR Safe (Invitrogen, Waltham, USA). The length of the amplified products was estimated using a 1 Kbp DNA ladder (Invitrogen) and the amplified products were visualized with an UV trans-illuminator (Bachofer, Germany), and photographed using a gel documentation system (BioDocAnalyze-Biometra Analytik GmbH, Göttingen, Germany).

### Cloning, expression, and purification of r*Bc*SBP4 protein

The full-length *sbp4* gene, amplified from DNA extracted using blood of a *B. caballi*-infected Egyptian donkey [6] as described above, was cloned into a TOPO^®^ TA vector (Invitrogen) following the manufacturer’s guidelines. The TOPO^®^ TA plasmid containing the full-length *sbp4* gene was transformed into competent *Escherichia coli* One Shot™ TOP10 cells (Invitrogen) and cultured in LBA media, according to the manufacture’s guidelines (Invitrogen). The *Bcsbp4* gene cloned into the TOPO^®^ plasmid was sequenced to confirm its identity.

For recombinant protein expression, the full-length *Bcsbp4* gene was cloned into the *pBAD* expression vector (Invitrogen) as described above, according to the manufacture protocol. The recombinant protein was purified using nickel columns. The recombinant protein was then eluted using pH-elution under denaturing conditions with urea eluting buffer (pH 4). The eluted recombinant protein was analyzed by SDS-PAGE and western blot using anti-histidine tag antibodies (Invitrogen). The purified r*Bc*SBP4 was quantified [16] and stored in aliquots at − 20 °C.

### Production of rabbit anti-SBP4 peptide sera and equid serum samples

Two synthetic peptides representing regions of the *Bc*SBP4 containing *in silico* predicted B-cell epitopes according to the method of Hopp & Woods [17] (AA253-271 C-DAFVAKREKLSAEAAEKSE; and AA117-128 GSPIHGKDGE-C) were generated (Additional file 2: Figure S1). An equimolar mix of the peptides was conjugated to KLH and used for producing antibodies in rabbits (Biosynthesis, Lewisville, Texas, USA). The rabbit was inoculated 4 times with 50 µg of KLH-conjugated peptides, administrated by the subcutaneous (SC) route every 2 weeks. The antibodies were prepared by Biosynthesis.

*Babesia caballi* positive horse sera as well as 10 negative horse sera samples, as determined by cELISA (VMRD) were obtained from USDA (USDA, Pullman, WA, USA) [8]. *Theileria equi* positive serum was obtained from experimentally infected horses from the USDA equine piroplasmosis laboratory (Pullman, WA, USA) [8]. All collected sera were stored at − 20 °C until used. The antibody against the 48 kDa *B. caballi* RAP-1 used in western blot analysis, was described previously [8]. The anti HAP2 peptide rabbit antibody used in western blot analysis was previously described [18].

### Western blot analysis

Lysates from *B. caballi in vitro* cultures infected RBCs were prepared as described previously, and fractionated using 10 % SDS-PAGE under reducing conditions [19]. After electrophoresis, gels were transferred to 0.45 nitrocellulose membranes according to Towbin et al. [20]. The nitrocellulose strips were incubated with 1:100 primary antibodies then incubated with protein A peroxidase conjugate at 1:2500 in 0.5% BSA/TBS buffer for 1 h. The immune-reactive bands were identified by incubation of the blot in the substrate solution (1-chloronaphthol) (Sigma-Aldrich, St. Louis, USA). The membranes were visualized using a gel documentation system (Bio-Rad, Hercules, USA).

### Localization of *Bc*SBP4 by immunofluorescence

The localization of *Bc*SBP4 by immunofluorescence was performed using glass slides prepared with acetone-fixed infected erythrocytes from *B. caballi in vitro* culture [8] blocked with 10% bovine serum albumin. Separate slides were incubated for 1 h with a 1/20 dilution using rabbit pre-immune serum, 1/20 dilution rabbit anti-SBP4 cocktail peptides, 1 μg/ml monoclonal antibody 79/17.18.5 reactive with *B. caballi* RAP-1, as previously described [8], used as a positive control, and 1 μg/ml monoclonal antibody Tryp1E1 that reacts with the *Trypanosoma brucei* variable surface glycoprotein used as negative isotype control as previously described [21]. After washing 3 times in 1× PBS, the cells were then incubated for 30 min using a 1/1000 dilution of goat anti-rabbit Alexa Fluor^®^ 647 or goat anti-mouse Alexa Fluor^®^ 488. After another 3 washes with 1× PBS, SlowFade^®^ gold antifade reagent with DAPI and a coverslip was applied, and the slides were visualized using a Leica DMI8 inverted fluorescent microscope to produce a merged image.

### Diagnostic immunofluorescence (IFA) and indirect ELISA (iELISA) analyses

Serological diagnosis of *B. caballi* infections was performed by IFA using infected blood slides kindly donated by VMRD Inc. (Pullman, WA, USA) as previously described [6]. *Babesia caballi* positive and negative control sera were donated by VMRD and used to evaluate an iELISA based on the r*Bc*SBP4 antigen with 1:100 dilution. A total of 18 horses and donkeys sera were screened using the r*Bc*SBP-4 iELISA for detection of specific antibodies against the *B. caballi* Egyptian isolate. The r*Bc*SBP-4 iELISA was optimized by a serial checkerboard titration as previously described [22]. ELISA plates (Greiner, Frickenhausen, Germany) were coated overnight at 4 °C with 100 μl of 0.2 μg/well of r*Bc*SBP-4 protein in 50 mM carbonate buffer, pH 9.6. After blocking with 2% dry skim-milk in coating buffer and washing with 0.01 M 0.05 % PBS-Tween 20 pH 7.4, the wells were incubated with 100 μl of diluted serum samples at 1:100 for 2 h at 37 °C. Then, plates were washed and incubated with 100 μl of diluted anti-horse IgG peroxidase conjugate (Sigma-Aldrich) at 1:2500 at 37 °C for 1 h. One hundred microliters of O-phenylenediamine solution (0.33 mg/ml in citrate buffer, pH 5.2, in the presence of 0.04 % hydrogen peroxide) were added and the reaction was stopped after 10–15 min by adding 20 µl of stop solution (0.16 M sulfuric acid). Positive, negative and no sample controls were tested in triplets, and the results were expressed as the mean of triplicate for each sample. Optical density (OD) was read at 405 nm using an ELISA plate reader (ELx800 UV; Bio-Tek Instruments, Winooski, USA). A serum sample was considered positive for specific antibody to *B. caballi* if it showed an OD higher than the mean plus 3 standard deviations of the negative serum samples.

### *In vitro* neutralization assay

The ability of anti-*Bc*SBP4 antibodies to neutralize *B. caballi* merozoite invasion of red blood cells (RBC) was evaluated *in vitro* using the anti-*Bc*SBP4 rabbit sera described above. The percentage of parasitized erythrocytes (PPE) were measured by flow cytometric analysis. Briefly, free merozoites were obtained from high parasitemia (> 40%) *B. caballi* cultures [8] by centrifugation at 500× *g*, 4 °C for 10 min. After that, free merozoites in the supernatant were counted using fluorescein diacetate and approximately 10^7^ free merozoites were incubated for 30 min at 37 °C with 1:10 dilution of sera. Pre-immune rabbit sera diluted 1:10 were used as negative controls and sera dilutions were tested in triplicate. Similarly, normal horse sera and *B. caballi*-infected horse (termed Ho A2034 Pre and Ho A2034 Pos, respectively) sera diluted 1:10 were used as negative and positive controls, respectively [8]. After incubation, merozoites were washed in Puck’s Saline G (Gibco, Aidenbach, Germany) and plated in 96-well plates with 5% horse RBC. The plates were kept at 37 °C 5% CO_2_ and PPE evaluated at days 2, 3 and 4 of culture. To calculate PPE in each time point, the cultures were washed in PBS, stained with hidroethidine (HE) (65 μM), and evaluated by flow cytometer. Flow cytometric analysis was performed using a Guava^®^ easyCyte flow cytometer (Luminex, Austin, USA), and data were acquired using InCyte (guavaSoft 3.1.1). A total of 20,000 events were collected for each sample and serum dilutions were tested in triplicates. After acquisition, results were analyzed in FCS Express version 6 (DeNovo™ Software; Pasadena, USA). RBCs were gated according to their complexity (SSC, side scatter) and size (FSC, forward scatter). HE stained cells were gated and quantified based on their fluorescence. Data are presented as PPE calculated by the mean of three experimental replicates. Means of PPE were compared with a two-tailed t-test using Prism 8 (GraphPad Software, San Diego, USA).

## Results and discussion

**Fig. 1:**
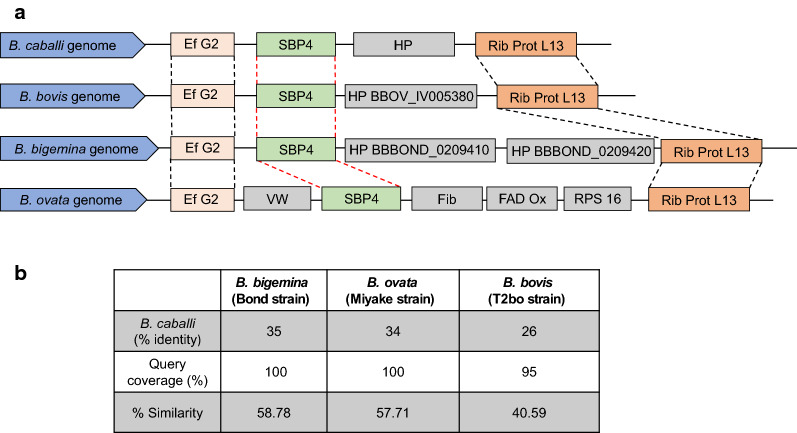
**a** Schematic representation for the synteny map framework of *B. caballi spb4* gene in comparison with its orthologous genes in the genomes of *B. bigemina*, *B. ovata* and *B. bovis*. *Babesia caballi* genome: Ef G2, Elongation factor G2; *B. bovis* Ef G2, Elongation factor G2 BBOV_IV005400; *B. bigemina* Ef G2, Elongation factor BBBOND_0209390; *B. ovata* Ef G2, Elongation factor BOVATA_011720; *B. ovata* VW, Von Willebrand factor type ABOVATA_011730; *B. bovis*, BBOV_IV00539; *B. bigemina*, BBBOND_0209400; *B. ovata*, BOVATA_011740; HP, hypothetical protein; *B. ovata* Fib, Fibronectin type III domain containing 3C1-like BOVATA_011750; *B. ovata* FAD Ox, FAD-dependent oxidoreductase BOVATA_011760; *B. ovata* RPS 16, Ribosomal protein RPS16 BOVATA_011770; *B. caballi* Rib, Protein L13, Ribosomal protein L13; *B. bovis* Rib Prot L13, Ribosomal protein L13 BBOV_IV005370; *B. bigemina* Ric Prot, Ribosomal protein L13BBBOND_0209430; *B. ovata* Rib L13, Ribosomal protein L13 BOVATA_011780. Schematic rectangles represent gene location and not gene size. **b** Percentages of amino acid sequence identity and similarity and query coverage among the SBP4 proteins of *B. caballi*, *B. bigemina*, *B. ovata* and *B. bovis*

The entire genome of the *B. caballi* Florida strain used in this study is currently being curated and assembled (Lowell Kappmeyer, personal communication). The search of the *B. caballi* genome assembly database using the *B. bovis* SBP4 sequence as a query (GenBank: XP_001610468.1) resulted in the identification of a *sbp4*-like gene, hereby named *Bcsbp4*. The *Bcsbp4* gene contains an 816 bp ORF encoding for a predicted protein of 30.58 kDa. Based on the *in silico* sequencing data, we designed primers and amplified the full-length *Bcsbp4* gene from DNA extracted from a *B. caballi*-infected donkey in Egypt. The *sbp4* gene derived from the USA (Puerto Rico strain) and Egyptian *B. caballi* isolates were sequenced and compared (GenBank: MT032179 and MT032180, respectively). A comparison among the fully identical SBP4 sequences derived from the USA and Egyptian isolates of *B. caballi* and the SBP4 of *B. bovis* is shown in Additional file 3: Figure S2a. Additional *in silico* analysis of the predicted *Bc*SBP4 protein revealed the presence of a 22-aa signal peptide and the absence of other transmembrane domains (Additional file 3: Figure S2b, Additional file 4: Figure S3). Overall, the general architecture of the *Bc*SBP4 resembles closely that of the previously characterized *B. bovis* SBP4 [13] (Fig. 1). We then compare sequences of *Bc*SBP4 with SBP4 proteins derived from other related *Babesia* parasites (Fig. 1). BLAST searches performed with the putative *Bc*SBP4 showed *e* scores that are very significant among the *B. bovis*, *B. bigemina*, and *B. ovata* SBP4s. In all cases, the *e* scores are in the range of 10^−30^ to 16^−63^ implying that the relationship between these proteins is extremely unlikely due to randomness, but instead, strongly suggest that they are all related. We found that *Bc*SBP4 is more similar to *B. bigemina* (35% identity), followed by the *B. ovata* (34% identity) SBP4 proteins, in comparison to other known *Babesia* SBP4 sequences (Fig. 1). In addition, to further determine that the *Bc*SBP4 is a real orthologue of the other known *Babesia* SBP4s we performed synteny analysis among the *sbp4* genes of *B. caballi*, *B. bovis*, *B. ovata*, and *B. bigemina* to determine whether these genes are positionally orthologous. We found that in all cases, the genomic environment of these *Babesia sbp4*s are similar (Fig. 1). Thus, the *sbp4s* genes of *B. bovis* and *B. caballi* are flanked by the same annotated genes (the Elongation factor G upstream to the SBP4 and ribosomal protein L13 in the downstream position) strongly suggesting that these are orthologous genes.

**Fig. 2:**
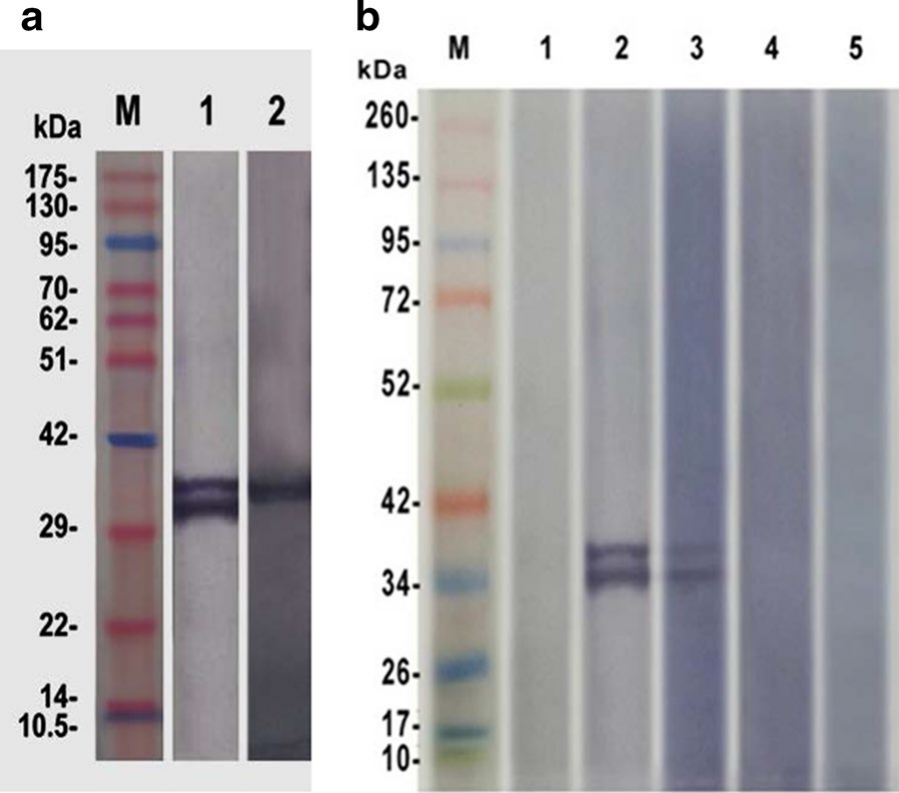
Western blot analysis. **a** Specificity of the rabbit immune sera generated against *Bc*SBP4 synthetic peptides. Lane 1: affinity purified r*Bc*-SBP-4; Lane 2: native *B. caballi* antigens derived from sonicated *in vitro* cultured parasites; Lane M: pre-stained molecular weight protein ladder. **b** The pattern of reactivity of r*Bc*-SBP4. Lane 1: pre-immune rabbit serum; Lane 2: rabbit immune sera generated against *Bc*-SBP4 synthetic peptides; Lane 3: sera from a *B. caballi*-infected horse; Lane 4: sera from a non-infected horse. Lane 5: sera from a *T. equi*-infected horse M: stained molecular weight protein ladder

Full-length *Bcsbp4*, from an Egyptian isolate, was amplified by PCR and cloned into the pBAD *E. coli* expression vector to generate r*Bc*SBP4. Western blot analysis of recombinant bacterial lysates using anti-histidine tag antibodies resulted in the detection of a 37 kDa recombinant protein (Additional file 5: Figure S4a). Two synthetic peptides representing *in silico* predicted regions of high immunogenicity in SBP4 (117-128aa; and 253-271aa) (Additional file 2: Figure S1) were synthetically produced, conjugated to KLH, and used to immunize rabbits. Antibodies derived from immunized rabbits were able to recognize recombinant and native SBP4 in western blot analysis (Fig. 2a). It is possible that the double band could be due to the presence of a truncated version of the recombinant protein, which may occur during the process of protein expression, or, it may also be due to protein degradation by a contaminating protease or the result of post-translational modifications in the prokaryotic expression system, or a combination of factors. In any case, these factors may result in a slower migrating of the recombinant protein in the SDS-PAGE gel. The presence of more than a single band in purified protein preparations is not uncommon during the process of production and purification of recombinant proteins produced in prokaryotic expression vectors. In addition, sera from a *B. caballi*-infected horse react specifically with the r*Bc*SBP4 (Fig. 2b) in western blot and indirect ELISA format (Additional file 5: Figure S4b). Furthermore, r*Bc*SBP4 did not react in immunoblots with sera from horses that were experimentally infected with *T. equi*. This pattern of reactivity is crucial if the antigen is a candidate for developing *B. caballi* specific serological diagnostic methods, especially considering that co-infections of equids with *T. equi* and *B. caballi* are frequent in endemic areas. Specificity of the anti *Bc*SBP4 peptide rabbit antibodies was demonstrated by western blot (Additional file 6: Figure S5).

**Fig. 3:**
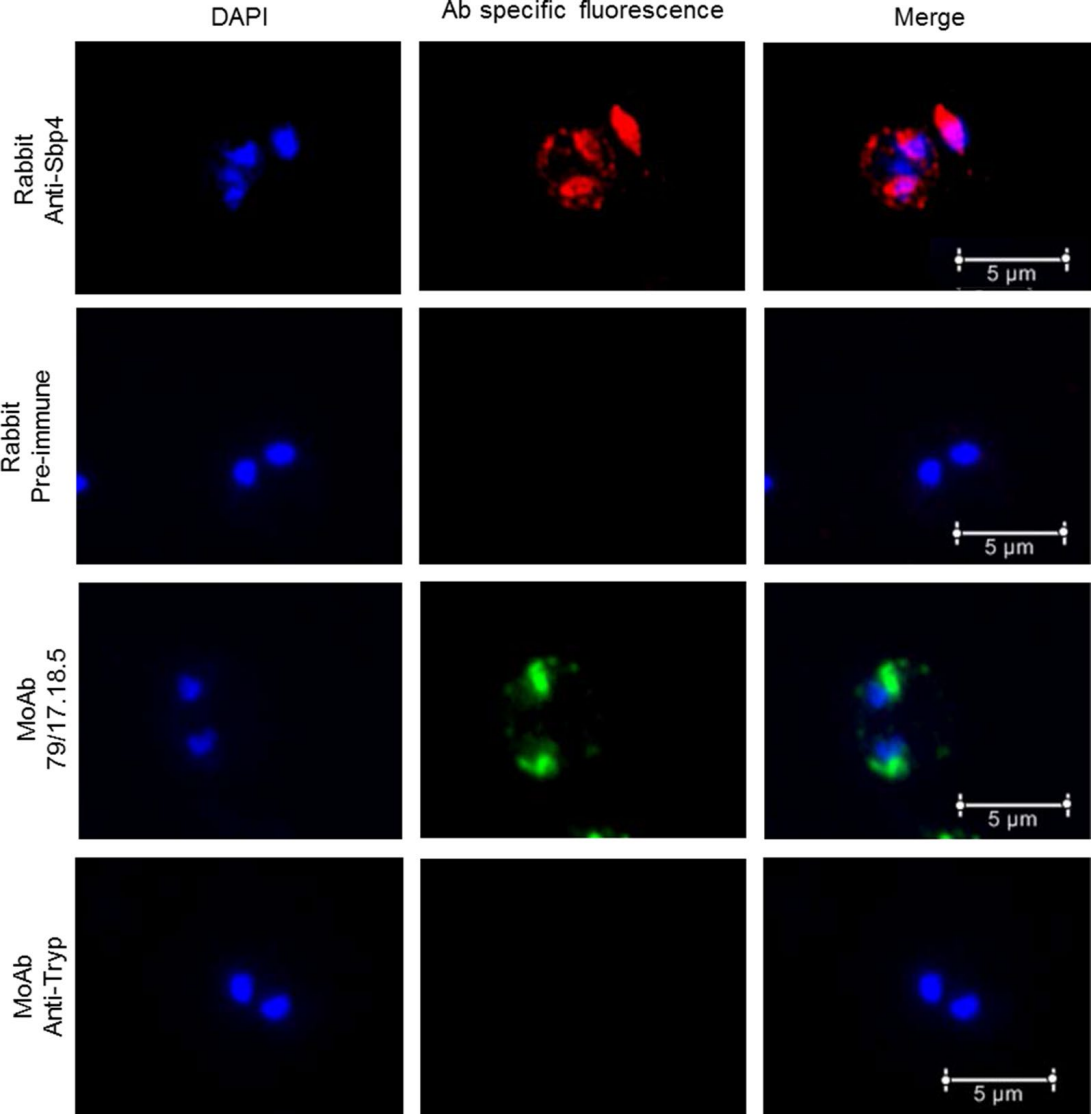
Immunofluorescence analysis of *in vitro* cultured *B. caballi* parasites with: rabbit anti-SBP4 cocktail peptides, rabbit pre-immune serum, monoclonal antibody 79/17.18.5 reactive with *B. caballi* RAP-1, and Tryp monoclonal irrelevant antibodies (the same isotype Ab as anti-RAP-1 79/17.18.5 mAb)

The pattern of expression and localization of SBP4 in *B. caballi*-infected erythrocytes was analyzed by IFA with the results shown in Fig. 3. The anti-*Bc*SBP4 peptide antibodies react with an antigen that appears to be localized in internal parasite organelles and also in the erythrocyte cytoplasm. This pattern of reactivity is compatible with spherical body localization and comparable to what was observed for other spherical body proteins in the related parasites *B. bovis*, *B. bigemina* and *B. orientalis* [13, 23, 24].

**Fig. 4:**
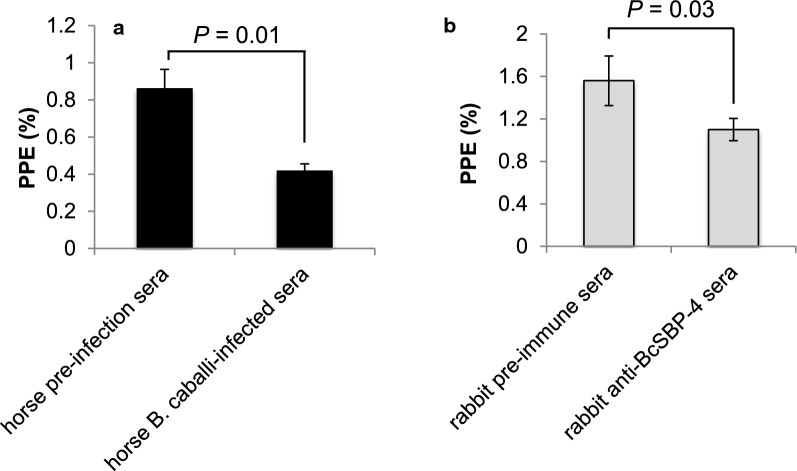
*In vitro* neutralization of *B. caballi* in *in vitro* cultures using sera from *B. caballi*-infected horses and from anti-Sbp4 rabbits. **a** Representation of the decrease in PPE at day 3 of *in vitro* cultured *B. caballi* merozoites in the presence of an immune *B. caballi* horse (Ho A2034) and pre-immune (Ho A2034 Pre) sera. **b** Anti-Sbp4 rabbit (rab 7082) and pre-immune control (rab7082 Pre) sera. *P* < 0.05 indicates a significant difference

We then investigated whether the anti-*Bc*SBP4 peptide antibodies were able to neutralize the infectivity of *B. caballi* merozoites in an *in vitro* culture test. While sera from a *B. caballi*-infected horse was able to considerably inhibit parasite growth in the *in vitro* cultures (48.8% average inhibition, *t*_(4)_ = 7.1601, *P* = 0.001), the anti-*Bc*SBP4 peptide antibodies had a marginal, although statistically significant, inhibitory effect (29.5% average inhibition, *t*_(4)_ = 3.1203, *P* = 0.035) which was evident only at day 3 after the addition of antibodies to the culture (*P* < 0.05) (Fig. 4a, b). The marginal difference found with the anti-*Bc*SBP4 antibodies (Fig. 4a, b) may reflect a functional importance of *Bc*SBP4. However, the relatively small impact of the antibodies in the growth of the parasite may be due to the intraerythrocytic expression pattern of *Bc*SBP4, as described for other highly related *Babesia* spherical body proteins [14]. This intra-host cell pattern of expression would likely prevent the interaction among *Bc*SBP4 and the anti-*Bc*SBP4 rabbit antibodies tested in the assay. However, it is also possible that *Bc*SBP4 is also expressed on the surface of free merozoites, and that the partial inhibition found may be due to some steric of functional hindrance caused by the binding of the anti-*Bc*SBP4 antibodies to such free merozoites. In addition, we have to also consider the possibility that the antibodies are raised against peptides that are not fully exposed in the surface of the parasites and thus unable to cause a stronger inhibitory effect on free merozoites (Fig. 4).

**Fig. 5:**
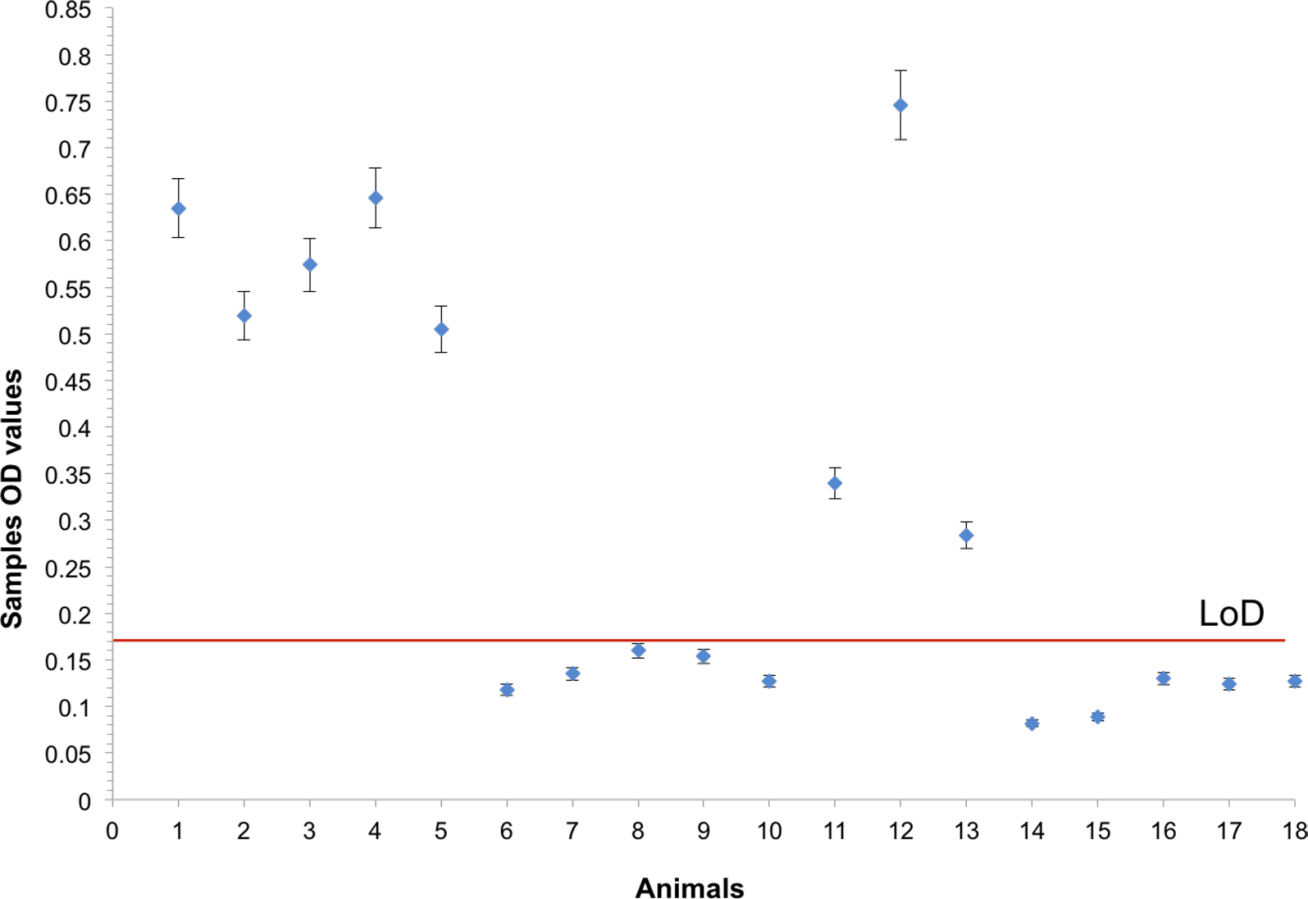
Detection of anti-*B. caballi* SBP4 antibodies in *B. caballi* equid infected sera using ELISA. Optical density (OD) values obtained in an ELISA analysis based on SBP4 recombinant protein, with a sample of sera from 18 equids. LoD - limit of detection calculated according to the formula x + 3*SD [0.1614]. Samples results under the LoD indicate negative results *vs* above the line indicates the positive results. Samples 1–10 are donkeys and 11–18 are horses

Next, we assessed whether *Bc*SBP4 is recognized by antibodies from *B. caballi*-infected horses and the potential use of r*Bc*SBP4 as an antigen in a diagnostic assay. To this end, we tested the reactivity of sera from eight horses and ten donkeys that were previously analyzed for *B. caballi* infection status using microscopy, PCR, and IFA, and in a r*Bc*SBP4-based iELISA (Table 1, Fig. 5). Using the IFA as a reference test, we found that all IFA negative sera animals were also identified as negative in the *Bc*SBP4-ELISA, whereas the sera from IFA positive equids tested positive. Samples 6, 7, 8, 10, 14, 16, 17 and 18 were negative regardless of the method of detection used, and likely represent non-infected or naïve animals. Interestingly, samples 9 and 15 were positive by film, but negative by any other method applied. Blood smears lack specificity and thus, it is possible that the parasites detected in these two samples are not actually *B. caballi*, but a related intraerythrocytic parasite, such as *T. equi*. Consistently, sera found positive using both serological methods were derived from animals that were also found positive either by microscopy and/or PCR (samples 2, 3, 4 and 11; Table 1). In addition, other samples that were positive in the IFA and ELISA tests, might correspond to *B. caballi*-infected equids that test negative in microscopy and PCR (samples 5, 12 and 13; Table 1). This may be due to very low parasitemia in peripheral blood, a typical feature occurring in *Babesia* persistent infections, or may belong to animals that clear their infections, either naturally or due to drug treatments. Samples 2 and 4 were both positive by film and IFA, and also in the ELISA test, but negative by PCR. It is likely that these samples belong to infected equids, but the DNA was either degraded, or contaminated by PCR reaction inhibitors. Alternatively, some of these cases are representative of animals that were able to fully clear the parasites from circulation, but still have circulating antibodies, and may be co-infected with intraerythrocitic parasites with a morphology similar to *B. caballi*. Overall, the performance of the r*Bc*SBP4 based iELISA is fully consistent with findings of the other three tests used in this study.

**Table 1:** Diagnosis data obtained from serum and blood samples collected from field-infected equids used in this study

Animal no.	Film	PCR	IFA	ELISA OD
1	–	+	+	0.635 (+)
2	+	–	+	0.519 (+)
3	–	+	+	0.574 (+)
4	+	–	+	0.646 (+)
5	–	–	+	0.505 (+)
6	–	–	–	0.118 (–)
7	–	–	–	0.135 (–)
8	–	–	–	0.16 (–)
9	+	–	–	0.154 (–)
10	–	–	–	0.127 (–)
11	–	+	+	0.34 (+)
12	–	–	+	0.746 (+)
13	–	–	+	0.284 (+)
14	–	–	–	0.082 (–)
15	+	–	–	0.089 (–)
16	–	–	–	0.13 (–)
17	–	–	–	0.124 (–)
18	–	–	–	0.127 (–)

*Abbreviations*: Film, Giemsa blood stain blood film slide; PCR, polymerase chain reaction; IFA, indirect immunofluorescence assay; ELISA, enzyme-linked immunosorbent assay; (–) indicates a negative result; (+) indicates a positive result

*Notes*: Samples 1–10 are donkeys and 11–18 are horses

## Conclusions

This study demonstrates the presence and expression of a gene in *B. caballi* that is similar to the *sbp4* genes in other *Babesia spp*. The *Bcsbp4* gene is expressed in erythrocyte stages of the parasite developed in *in vitro* cultures and, as demonstrated by its reactivity with antibodies from infected animals. The gene is also expressed and immunogenic during *B. caballi* infection of horses and donkeys. Importantly, the *sbp4* gene appears to be highly conserved among two diverse geographic strains of *B. caballi*. Taken together, the immunoblot and iELISA data suggest that the *Bc*SBP4 protein is a potential antigen for the development of improved serological methods for the detection of *B. caballi*. This *Bc*SBP4-based novel assay could be an alternative approach to other available tests that were found to be ineffective for the diagnostic of *B. caballi* infection in the Middle East, including Egypt, and globally. Further work is needed to assess conservation of *Bc*SBP4 among diverse strains and test the suitability of an iELISA based on r*Bc*SBP4 as a global diagnostic tool for the detection of *B. caballi* in infected equids. Alternatively, *Bc*SBP4 can also become a useful antigen for the development of other serological diagnostic tests, such as the modified indirect ELISA (MI-ELISA) as previously described for *B. bovis* [25], or in other formats, including immunochromatographic tests.

## Supplementary information

**Additional file 1: Dataset S1.** Amino acid sequence of *Bc*SPB4.

**Additional file 2: Figure S1.** Features of the *Bc*SBP4 protein: the *Bc*SBP4 was predicted to be secreted. The location of the predicted signal peptide and the regions containing the two peptides selected for antibody production are indicated by a green rectangle. The predictions were performed using online software at http://wlab.ethz.ch/protter.

**Additional file 3: Figure S2. a** Sequence alignment among the deduced SBP-4 amino acid sequences from Egypt and USA *B. caballi* isolates (1, 2) and SBP4 from *Babesia bovis* (3). Consensus sequences are shown in the bottom row. **b***In silico* prediction of trans-membrane domains and signal peptides in Egyptian *B. caballi* SBP-4 using TMHMM2.

**Additional file 4: Figure S3.** Secondary structure prediction of *B. caballi* SBP4 (http://minnou.cchmc.org/).

**Additional file 5: Figure S4. a** Anti-histidin antibodies recognize a 37 kDa antigen in a cell lysate of recombinant *E. coli* transformed with TOPO® TA plasmid containing the full-length *Bcsbp4* gene. Lane 1: (+ ve) cultures induced with L-Arabinose; Lane 2: (- ve) non-Arabinose induced cultures. **b** Specific recognition of r*Bc*SBP4 protein by *B. caballi* positive serum in ELISA (a 1:100 dilution of serum was serially diluted by two-fold) using: serum from an equid derived from a horse experimentally infected with *B. caballi* (blue columns); field horse positive for *B. caballi* infection (orange columns); and horse that tested negative to infection with *B. caballi* (grey columns).

**Additional file 6: Figure S5.** Specificity of the anti *Bc*SBP4 peptide rabbit antibodies: western blot analysis against (i) normal horse RBC lysate, (ii) *B. caballi-*infected RBC lysate, (iii) recombinant *B. caballi* Spb4 and (iv) recombinant *B. bovis* Hap2 using serum indicated above. *Abbreviation*: M, size markers.

## Data Availability

Data supporting the conclusions of this article are included within the article and its additional files. The datasets used and/or analyzed during the present study are available from the corresponding author upon reasonable request.

## Abbreviations

SBP4: spherical body protein 4
r*Bc*SBP4: recombinant *Babesia cabali* spherical body protein 4
IFA: immunofluorescence assay
