# Supplementary material for: Identification and antigenicity of the Babesia caballi spherical body protein 4 (SBP4)
Source: Parasit Vectors. 2020 Jul 22;13:369. doi: 10.1186/s13071-020-04241-9 (PMC7376649; doi:10.1186/s13071-020-04241-9)
Supplement: Supplementary file 1 — Additional file 1: Dataset S1. Amino acid sequence of BcSPB4. [file 13071_2020_4241_MOESM1_ESM.pdf]

>*B. caballi* SPB4 protein sequence

MAAFSTRSLLLTCVLSVAGALAFAPFYEPNYQLHGNPDAMVLDILKPVNRSLIRKEVLE
CGARVPVYFTPVKPALLRGVYWGSNLIFGVDEDSAETIEKVTVYRNCYNSLVIVSVGGS
PIHFHGKDGEFEIISEEGFGEELEKMDRQTTVNVSQRATNDFVVVEEDRCFNLPWRNILP
ARCFQSDKVMDNEIPIWKARQCSERFAGATAFDDGEQKVVAVVVRDDAEVKEFFYHS
KGECYHEITAEEFRSFYDAFVAKREKLSAEAAEKSE
