# Supplementary figures and images for: Identification and antigenicity of the Babesia caballi spherical body protein 4 (SBP4)
Source: Parasit Vectors. 2020 Jul 22;13:369. doi: 10.1186/s13071-020-04241-9 (PMC7376649; doi:10.1186/s13071-020-04241-9)

## Slide 1
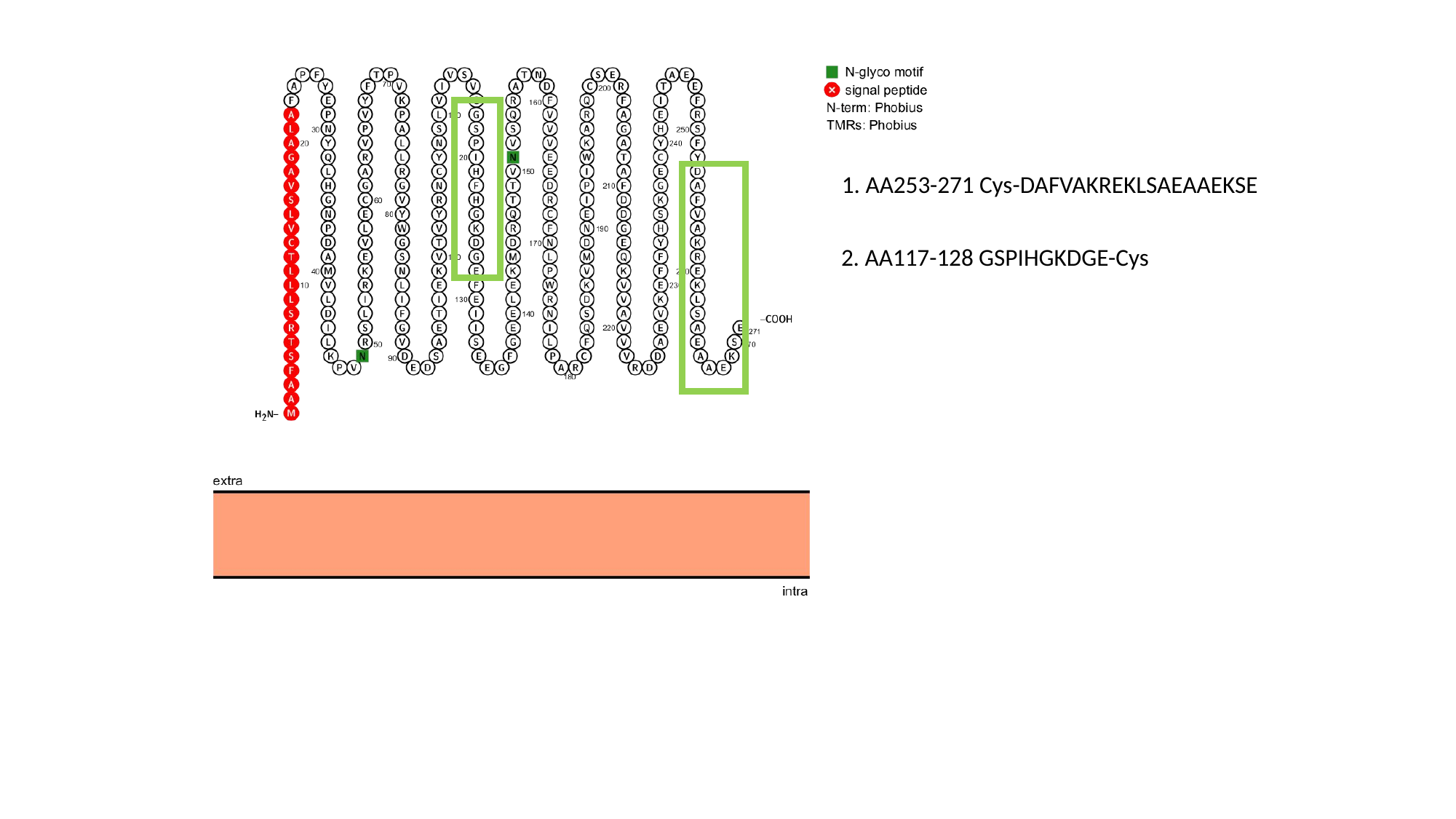

1. AA253-271 Cys-DAFVAKREKLSAEAAEKSE
2. AA117-128 GSPIHGKDGE-Cys

Supplement: Supplementary file 2 — Additional file 2: Figure S1. Features of the BcSBP4 protein: the BcSBP4 was predicted to be secreted. The location of the predicted signal peptide and the regions containing the two peptides selected for antibody production are indicated by a green rectangle. The predictions were performed using online software at http://wlab.ethz.ch/protter. [file 13071_2020_4241_MOESM2_ESM.pptx]

## Slide 1
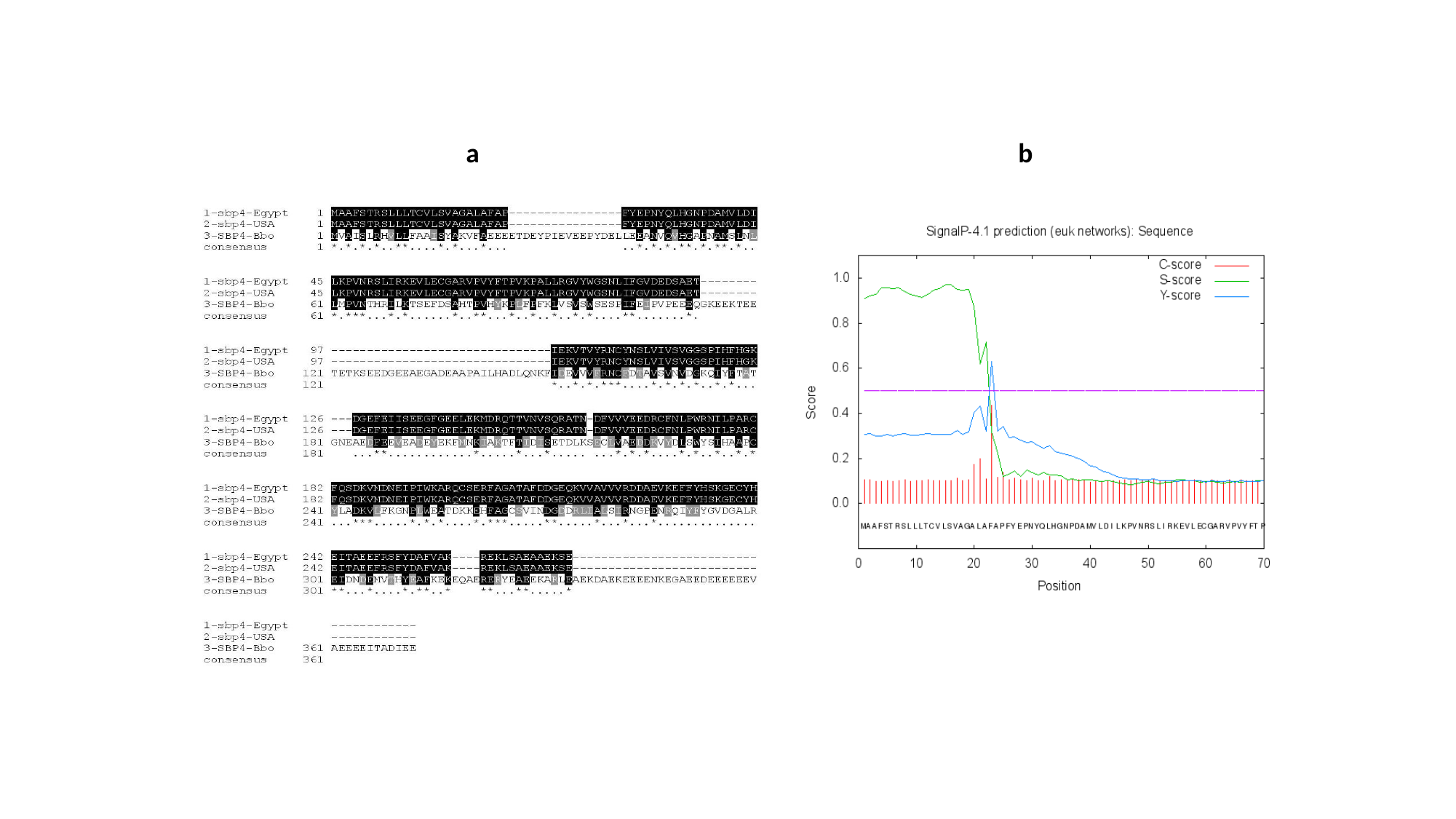

a
b

Supplement: Supplementary file 3 — Additional file 3: Figure S2. a Sequence alignment among the deduced SBP-4 amino acid sequences from Egypt and USA B. caballi isolates (1, 2) and SBP4 from Babesia bovis (3). Consensus sequences are shown in the bottom row. bIn silico prediction of trans-membrane domains and signal peptides in Egyptian B. caballi SBP-4 using TMHMM2. [file 13071_2020_4241_MOESM3_ESM.pptx]

## Slide 1
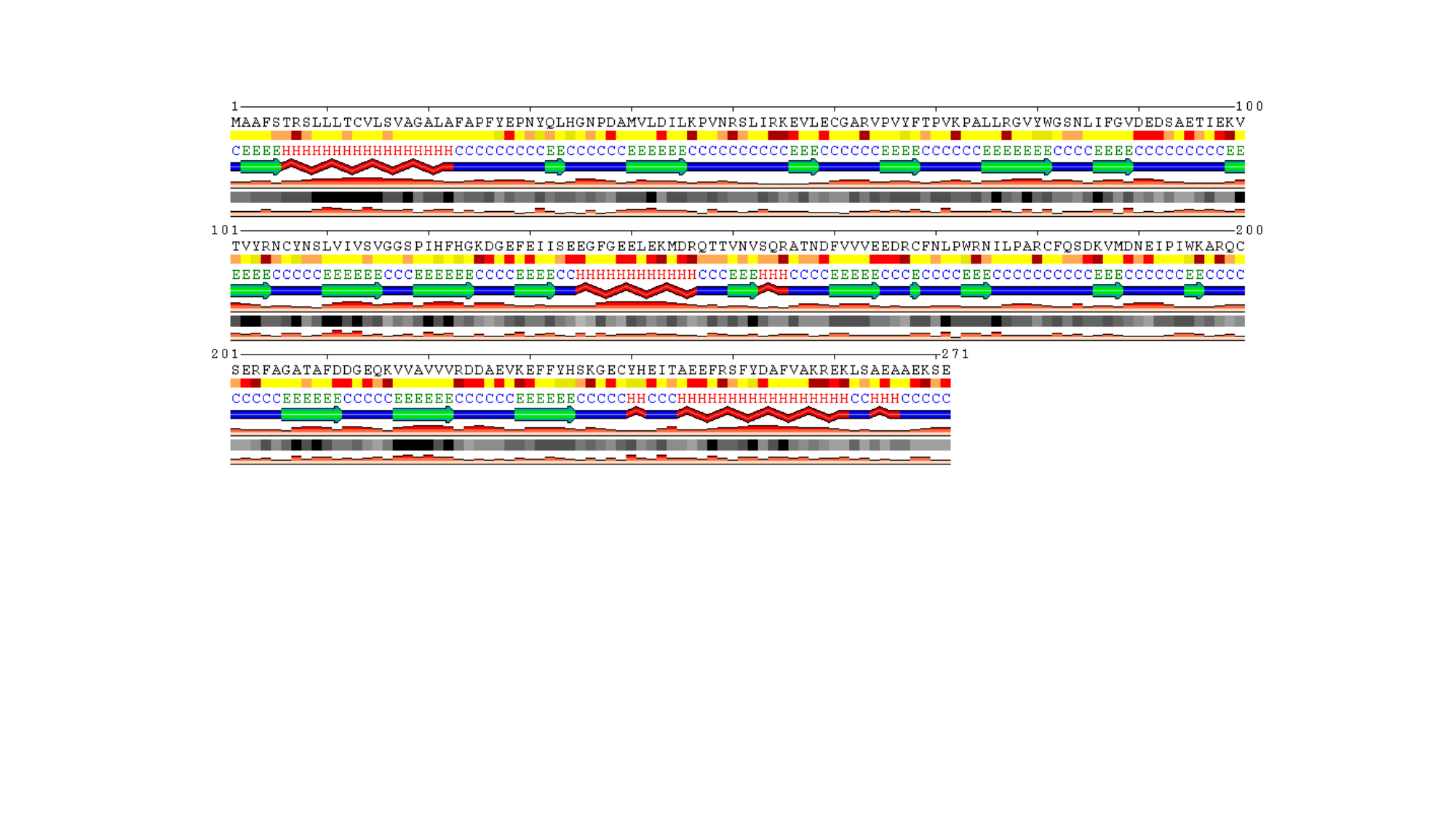

Supplement: Supplementary file 4 — Additional file 4: Figure S3. Secondary structure prediction of B. caballi SBP4 (http://minnou.cchmc.org/). [file 13071_2020_4241_MOESM4_ESM.pptx]
