# Supplementary material for: Identification and antigenicity of the Babesia caballi spherical body protein 4 (SBP4)
Source: Parasit Vectors. 2020 Jul 22;13:369. doi: 10.1186/s13071-020-04241-9 (PMC7376649; doi:10.1186/s13071-020-04241-9)
Supplement: Supplementary file 5 — Additional file 5: Figure S4. a Anti-histidin antibodies recognize a 37 kDa antigen in a cell lysate of recombinant E. coli transformed with TOPO® TA plasmid containing the full-length Bcsbp4 gene. Lane 1: (+ ve) cultures induced with L-Arabinose; Lane 2: (- ve) non-Arabinose induced cultures. b Specific recognition of rBcSBP4 protein by B. caballi positive serum in ELISA (a 1:100 dilution of serum was serially diluted by two-fold) using: serum from an equid derived from a horse experimentally infected with B. caballi (blue columns); field horse positive for B. caballi infection (orange columns); and horse that tested negative to infection with B. caballi (grey columns). [file 13071_2020_4241_MOESM5_ESM.pptx]

## Slide 1
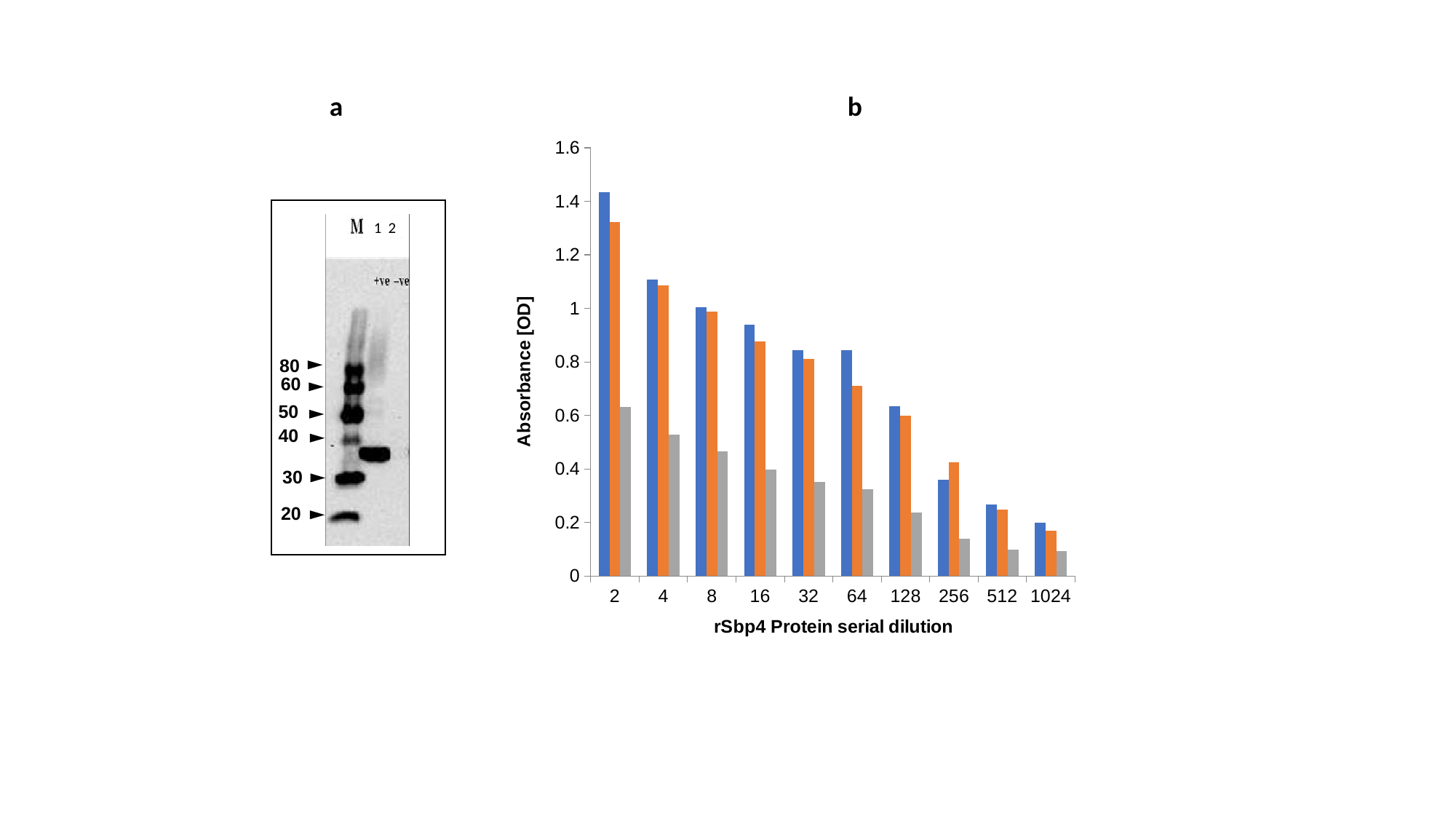

a
b
### Chart
| Category | Positive serum B.cabali [expermintal infection] | Positive serum from field infected animal diagnosed by IFA | Negative horse sera |
|---|---|---|---|
| 2 | 1.433 | 1.322 | 0.631 |
| 4 | 1.107 | 1.086 | 0.528 |
| 8 | 1.003 | 0.987 | 0.465 |
| 16 | 0.938 | 0.876 | 0.398 |
| 32 | 0.845 | 0.81 | 0.351 |
| 64 | 0.844 | 0.711 | 0.324 |
| 128 | 0.633 | 0.6 | 0.238 |
| 256 | 0.359 | 0.424 | 0.139 |
| 512 | 0.267 | 0.249 | 0.099 |
| 1024 | 0.198 | 0.169 | 0.094 |
1 2
80
60
50
40
30
20
