# Supplementary material for: Identification and antigenicity of the Babesia caballi spherical body protein 4 (SBP4)
Source: Parasit Vectors. 2020 Jul 22;13:369. doi: 10.1186/s13071-020-04241-9 (PMC7376649; doi:10.1186/s13071-020-04241-9)
Supplement: Supplementary file 6 — Additional file 6: Figure S5. Specificity of the anti BcSBP4 peptide rabbit antibodies: western blot analysis against (i) normal horse RBC lysate, (ii) B. caballi-infected RBC lysate, (iii) recombinant B. caballi Spb4 and (iv) recombinant B. bovis Hap2 using serum indicated above. Abbreviation: M, size markers. [file 13071_2020_4241_MOESM6_ESM.pptx]

## Slide 1
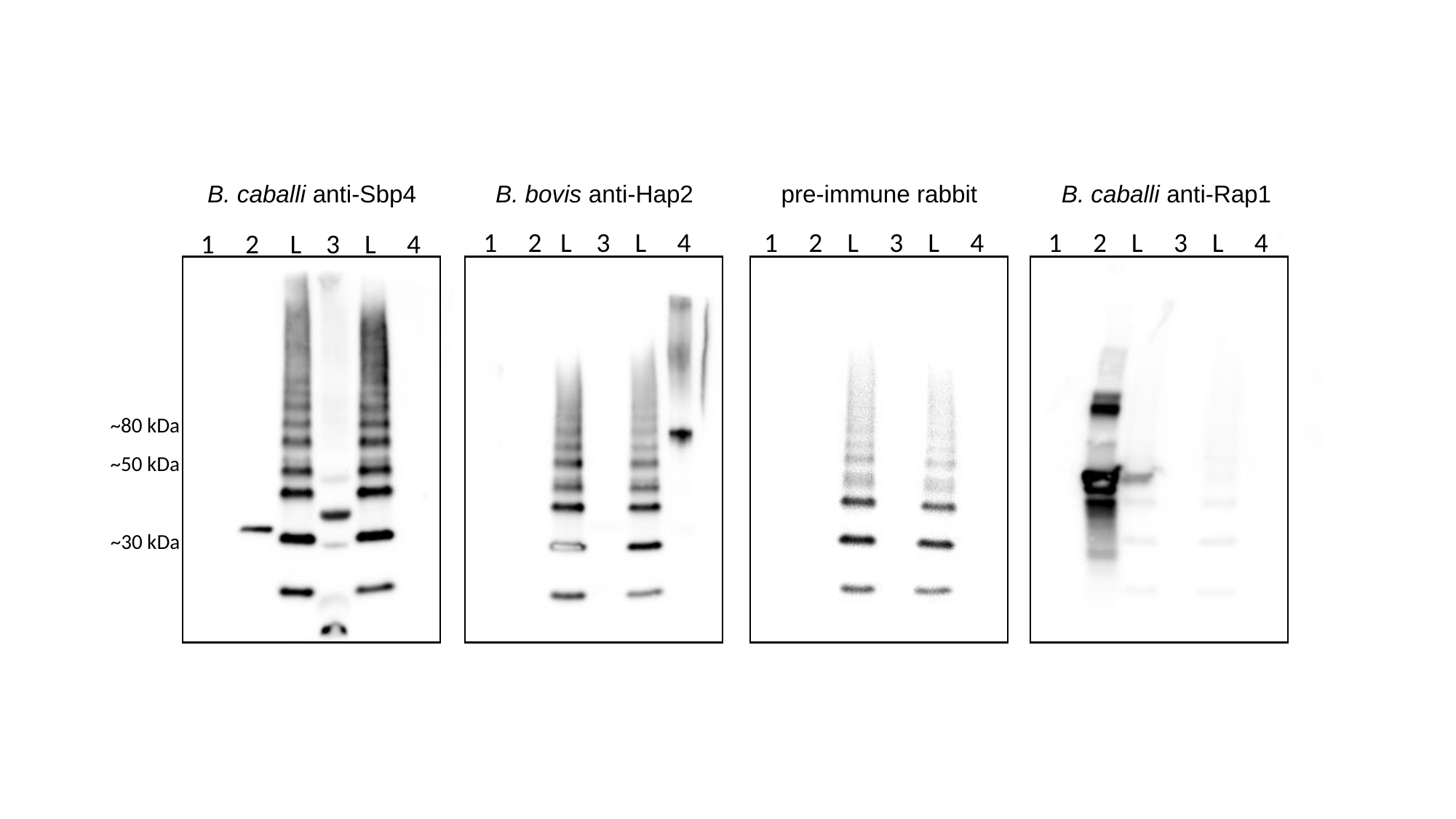

B. caballi anti-Sbp4
B. bovis anti-Hap2
pre-immune rabbit
B. caballi anti-Rap1
 1 2 L 3 L 4
 1 2 L 3 L 4
 1 2 L 3 L 4
 1 2 L 3 L 4
~80 kDa
~50 kDa
~30 kDa
